# Preprocessing of ^18^F-DMFP-PET Data Based on Hidden Markov Random Fields and the Gaussian Distribution

**DOI:** 10.3389/fnagi.2017.00326

**Published:** 2017-10-09

**Authors:** Fermín Segovia, Juan M. Górriz, Javier Ramírez, Francisco J. Martínez-Murcia, Diego Salas-Gonzalez

**Affiliations:** ^1^Department of Signal Theory, Networking and Communications, University of Granada, Granada, Spain; ^2^Department of Psychiatry, University of Cambridge, Cambridge, United Kingdom

**Keywords:** PET image segmentation, ^18^F-DMFP-PET data, intensity normalization, Hidden Markov Models, Gaussian distribution, Parkinson's disease

## Abstract

^18^F-DMFP-PET is an emerging neuroimaging modality used to diagnose Parkinson's disease (PD) that allows us to examine postsynaptic dopamine *D*_2/3_ receptors. Like other neuroimaging modalities used for PD diagnosis, most of the total intensity of ^18^F-DMFP-PET images is concentrated in the striatum. However, other regions can also be useful for diagnostic purposes. An appropriate delimitation of the regions of interest contained in ^18^F-DMFP-PET data is crucial to improve the automatic diagnosis of PD. In this manuscript we propose a novel methodology to preprocess ^18^F-DMFP-PET data that improves the accuracy of computer aided diagnosis systems for PD. First, the data were segmented using an algorithm based on Hidden Markov Random Field. As a result, each neuroimage was divided into 4 maps according to the intensity and the neighborhood of the voxels. The maps were then individually normalized so that the shape of their histograms could be modeled by a Gaussian distribution with equal parameters for all the neuroimages. This approach was evaluated using a dataset with neuroimaging data from 87 parkinsonian patients. After these preprocessing steps, a Support Vector Machine classifier was used to separate idiopathic and non-idiopathic PD. Data preprocessed by the proposed method provided higher accuracy results than the ones preprocessed with previous approaches.

## 1. Introduction

Neuroimaging data have become an essential tool to diagnose the most frequent neurodegenerative disorders: Alzheimer's and Parkinson's disease. Initially, the neuroimages were visually inspected by experienced clinicians in order to corroborate a previous tentative diagnosis based on neuropsychological and behavioral tests. To this end, they looked for areas of low activation located in specific brain regions that are known to be affected by the supposed disorder. During the last decade, the neuroimaging community has progressively increased the use of computer toolboxes to analyze neuroimaging data (Friston et al., 2006; Schrouff et al., 2013). These tools are able to carry out statistical analyses that perform a more exhaustive examination of the huge amounts of information contained in the data and remove the subjectivity inherent to the visual inspection of the neuroimages (Górriz et al., 2017). However, these statistical analyses require additional preprocessing steps that make neuroimages from different subjects comparable. Two procedures are usually performed: spatial registration and intensity normalization (Saxena et al., 1998; Dukart et al., 2013). The former ensures that a given voxel from different neuroimages corresponds to the same anatomical position while the latter removes the differences due to the scanner used or the amount of radiopharmaceutical injected (Salas-Gonzalez et al., 2013). Even when all the data are acquired using a single scanner, an intensity normalization of the data is desirable. Several studies suggested that the absolute values of cerebral blood flow and other metabolic measurements have a coefficient of variance about 15% in healthy elderly subjects and as high as 30% in patients suffering neurodegenerative disorders (Leenders et al., 1990; Huang et al., 2007; Borghammer et al., 2009). In addition to spatial registration and intensity normalization, a segmentation step can be carried out. This procedure consists on partitioning the data into two or more maps each one containing information of different classes. For example, brain Magnetic Resonance Imaging (MRI) data are usually segmented into gray matter, white matter and cerebrospinal fluid. Segmentation is common in studies that use structural data but it has been also used for functional data. In Moussallem et al. (2012) the authors used a threshold (adjusted by an *ad hoc* function) to segment ^18^F-FDG-PET data in order to delimit tumors. A more sophisticated approach for the same purpose was demonstrated in Li et al. (2017).

^18^F-DMFP-PET is a neuroimage modality that is increasingly being used as an effective tool to distinguish between idiophatic and non-idiophatic parkinsonian patients and therefore to assist the diagnosis of Parkinson's disease (PD) (la Fougère et al., 2010). In contrast to DaTSCAN (widely used for PD diagnosis; Towey et al., 2011; Illán et al., 2012; Segovia et al., 2012;Martínez-Murcia et al., 2014), ^18^F-DMFP-PET is able to image the postsynaptic striatal dopaminergic deficit that characterizes non-idiopathic parkinsonian variants such as multiple system atrophy (MSA) or progressive supranuclear palsy (PSP). Because of this, most of the studies with ^18^F-DMFP-PET are focused on analyzing the striatal region, even though this neuroimaging modality contains moderate signal intensities in regions other than the striatum that can be useful in PD diagnosis (Segovia et al., 2015, 2017).

In this work, we propose a methodology to preprocess ^18^F-DMFP-PET data that improves the results of subsequent analyses. The proposed method consists of two steps: data segmentation using Hidden Markov Random Fields (HMRF) and intensity normalization using the Gaussian distribution. The segmentation step divides each neuroimage into 4 maps: (i) high-signal voxels (located in the striatum), (ii) medium-signal voxels (located in most of the regions other than the striatum), (iii) low-signal voxels (most of then correspond to the cerebrospinal fluid), and (iv) voxels with intensities around zero (located outside the brain). The second step normalizes the intensities of each map using a Gaussian model. This approach was evaluated and compared with previous approaches using 87 neuroimages and a system based on Support Vector Machine (SVM) classification (Vapnik, 2000). The obtained results suggest that our procedure improves the automatic separation of idiopathic and non-idiopathic parkinsonian patients. In addition, it allows us to independently analyze the striatum and the remaining regions of the brain.

## 2. Materials and methods

### 2.1. Ethics statement

Each patient (or a close relative) gave written informed consent to participate in the study and the protocol was accepted by the Ethics Committee of the University of Munich. All the data were anonymized by the clinicians who acquired them before being considered in this work.

### 2.2. ^18^F-DMFP-PET neuroimaging database

Eighty-seven ^18^F-DMFP-PET neuroimages were used to evaluate the preprocessing approach proposed in this work. These data were collected during a longitudinal study carried out by the University of Munich (la Fougère et al., 2010). The neuroimages were acquired 55 min after the ^18^F-DMFP injection (which was synthesized using an automatic synthesis module as described in la Fougère et al., 2010) by means of a Siemens/CTI camera. Neuroleptics, metoclopramide and other medications and dopamine agonists that could potentially interfere were withdrawn before the data acquisition according to their biologic half-life. The emission recording consisted of 3 frames of 10 min each, acquired in 3-dimensional mode. The resulting images were reconstructed as 128 × 128 matrices of 2 × 2 mm voxels by filtered backprojection using a Hann filter.

All the patients included in the study were referred to ^18^F-DMFP-PET examination from local movement disorder clinics. They showed parkinsonian movement disorders and nigrostriatal degeneration that were confirmed by a ^123^I-FP-CIT SPECT scan according to widely accepted criteria (Koch et al., 2005). They were monitored during 2 years after the ^18^F-DMFP-PET acquisition and at this time the neuroimaging data were labeled according to the last diagnosis. Specifically, the last diagnosis was based on the response to an apomorphine challenge test or the response to dopamine replacement therapy and follow-up clinical examinations, paying special attention to orthostatic hypotension, cerebellar signs, eye movement disorders, spasticity or other atypical symptoms. Table 1 shows the resulting groups and some demographic details.

**Table 1:** Group distribution of the neuroimaging data considered in this work (*μ* and *σ* stand for the mean and the standard deviation, respectively).

		**Sex**		**Age**		
	**#**	**M**	**F**	**μ**	**σ**	**Range**
PD	39	22	17	61.38	11.14	35–81
MSA	24	20	4	68.42	10.73	43–85
PSP	24	12	12	69.29	7.33	55–84

Before applying the proposed method and the subsequent classification, the data were spatially registered using the template matching algorithm implemented in Statistical Parametric Mapping (SPM) (Friston et al., 2006). This procedure makes each neuroimage matches a given template, pursuing the same position (in the neuroimage space) in different neuroimages corresponds to the same anatomical position. The template was computed as follows: first all the neuroimages were registered to a randomly chosen one. The registered images and their hemisphere midplane reflections were then averaged (this step ensured a symmetric template). Finally the resulting image was smoothed and used to register the whole dataset (Ashburner et al., 1997).

### 2.3. Markov models

A Markov model (a.k.a. Markov chain) is a discrete stochastic process in which the next state only depends on the current state. If unobserved (hidden) states are assumed, the model is known as hidden Markov model (HMM). This work is focused on Markov random fields (MRF) that can be considered a generalization of Markov models for multiple-dimensions problems.

#### 2.3.1. Markov random fields

Markov random field theory is a branch of probability theory for analyzing the spatial or contextual dependencies of physical phenomena. A MRF is a family of random variables that satisfies the Markovianity property and can be described by an undirected graphical model.

Let *S* = {1, 2, …, *N*} be the set of indexes in space, and $N=\left\{N_{i},i\in S\right\}$ a neighborhood system, with $N_{i}$ being the set of sites neighboring *i* and satisfying that $i\notin N_{i}$ and $i\in N_{j}\Leftrightarrow j\in N_{i}$. A random field is said to be a MRF on S with respect to a neighborhood system N if and only if Li (2001):

(1) $$\begin{array}{r} \begin{array}{c} P\left(\text{x}\right)>0,\forall \text{x}\in \chi \\ P\left(x_{i}|x_{S-\left\{i\right\}}\right)=P\left(x_{i}|x_{N_{i}}\right) \end{array} \end{array}$$

where **x** = (*x*_1_, *x*_2_, …, *x*_*N*_) is a configuration in *S* and χ is the set of all possible configurations in *S*. A MRF can be characterized by a Gibbs distribution, allowing us to redefine the probability *P*(**x**) as (Hammersley-Clifford theorem):

(2) $$\begin{array}{r} P\left(\text{x}\right)=\frac{1}{Z}\exp\left(-T^{-1}U\left(\text{x}\right)\right) \end{array}$$

where:

(3) $$\begin{array}{r} Z=\underset{\text{x}\in \chi }{\sum }\exp\left(-T^{-1}U\left(\text{x}\right)\right) \end{array}$$

is a normalizing constant, *T* is a constant called temperature and usually fixed to 1 and *U*(**x**) is the energy function, defined as a sum of clique potentials *V*_*c*_(**x**) over all possible cliques, *C*:

(4) $$\begin{array}{r} U\left(\text{x}\right)=\underset{c\in C}{\sum }V_{c}\left(\text{x}\right) \end{array}$$

In this context, a clique *c* for the graph constituted by *S* and N (*S* contains the nodes and N the links) is defined as a subset of *S* whose elements are neighbors to one another (Li, 2001).

#### 2.3.2. Hidden markow random fields

Hidden Markow random fields are a generalization of HMMs that assume MRFs (more than one dimension) instead of Markov models (one dimension) and therefore, they can be directly applied to two and three-dimensional problems, such as neuroimage segmentation.

A HMRF is characterized by an unobservable (hidden) MRF *X* = {*X*_*i*_, *i* ∈ *S*} assuming values in a finite state space *L*, an observable random field *Y* = {*Y*_*i*_, *i* ∈ *S*} assuming values in a finite state space *D*, and a conditional independence restriction (Zhang et al., 2001). For any particular configuration **x** ∈ χ, every *Y*_*i*_ follows a known conditional probability distribution *p*(*y*_*i*_|*x*_*i*_) of the same functional form *f*(*y*_*i*_; *θ*_*x*_*i*__). Given that (conditional independence):

(5) $$\begin{array}{r} P\left(\text{y}|\text{x}\right)=\underset{i\in S}{\prod }P\left(y_{i}|x_{i}\right) \end{array}$$

the joint probability of (*X, Y*) can be written as:

(6) $$\begin{array}{r} P\left(\text{y},\text{x}\right)=P\left(\text{y}|\text{x}\right)P\left(\text{x}\right)=P\left(\text{x}\right)\underset{i\in S}{\prod }P\left(y_{i}|x_{i}\right) \end{array}$$

Since $P\left(y_{i},x_{i}|x_{N_{i}}\right)=P\left(y_{i}|x_{i}\right)P\left(x_{i}|x_{N_{i}}\right)$ (because of the local characteristics of MRFs), the marginal probability distribution of *Y*_*i*_ can be computed in function of the parameter set *θ* and $X_{N_{i}}$:

(7) $$\begin{array}{r} p\left(y_{i}|x_{N_{i}},\theta \right)=\underset{l\in L}{\sum }p\left(y_{i},l|x_{N_{i}},\theta \right)=\underset{l\in L}{\sum }f\left(y_{i};\theta_{l}\right)p\left(l|x_{N_{i}}\right) \end{array}$$

### 2.4. Automatic segmentation based on HMRF

The segmentation based on HMRF assigns a label *l*_*i*_ ∈ *L* = {1, 2, 3, 4}, *i* = {1, …*N*} to each voxel in a ^18^F-DMFP-PET neuroimage according to both intensity and neighborhood. Let **y** = {*y*_1_, *y*_2_, …, *y*_*N*_} be the intensity levels of the *N* voxels that form a ^18^F-DMFP-PET neuroimage. In this procedure we looked for a labeling **x** = (*x*_1_, *x*_2_, …, *x*_*N*_), where *x*_*i*_ ∈ *L* is the label assigned to the voxel *y*_*i*_. Formally we estimated (MAP criterion):

(8) $$\begin{array}{r} \hat{\text{x}}=\underset{\text{x}\in \chi }{arg max}\left\{P\left(\text{y}|\text{x}\right)P\left(\text{x}\right)\right\} \end{array}$$

where $\hat{\text{x}}$ is an estimation of **x** and considered a particular realization of the MRF *X*. Using the equivalence between MRFs and Gibb distributions the Equation (8) can be written as Zhang et al. (2001):

(9) $$\begin{array}{r} \hat{\text{x}}=\underset{\text{x}\in \chi }{arg min}\left\{U\left(\text{y}|\text{x}\right)+U\left(\text{x}\right)\right\} \end{array}$$

where *U*(**y**|**x**) is the likelihood energy. Estimating $\hat{\text{x}}$ involves estimating the parameter set *θ* = {*θ*_*l*_, *l* ∈ *L*}, where *θ*_*l*_ = (μ_*l*_, σ_*l*_), since we assumed a Gaussian function for each of the maps resulting from the segmentation of **y**. A *k*-means algorithm was used to initialize the labeling $\hat{\text{x}}$. Then, an Expectation-Maximization (EM) algorithm was carried out to alternatively estimate the parameter set, *θ*, and the label set, $\hat{\text{x}}$.

**Figure 1:**
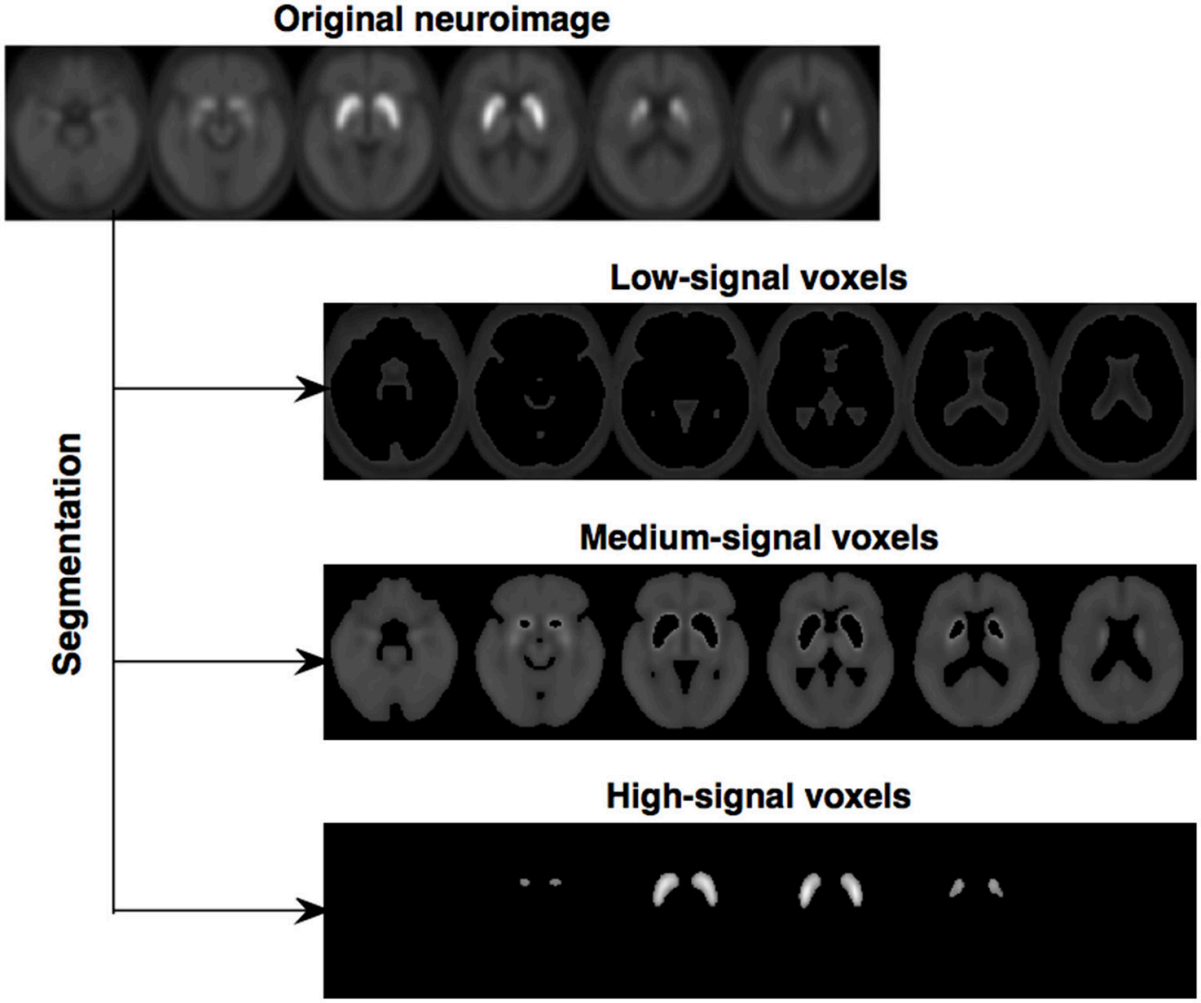
Segmentation of the image computed as the average of all the ^18^F-DMFP-PET neuroimages (the map with the voxels outside the brain was not represented). Note that two regions of interest, containing medium-signal and high-signal voxels, are clearly delimited.

Altogether, this segmentation procedure divides a neuroimage into 4 maps: (i) voxels with intensity close to zero (mainly voxels outside the brain), (ii) low-signal voxels, with very limited diagnostic value, (iii) medium-signal voxels, and (iv) high-signal voxels, with high diagnostic value (located in the striatal region). In order to reduce the computational burden, the segmentation procedure was only applied to an *ad-hoc* neuroimage computed as the average of all the ^18^F-DMFP-PET images in our dataset (the result is shown in Figure 1). Then, the resulting maps were used as binary masks to segment the neuroimages in our dataset.

In this initial work, only striatal voxels were considered to separate idiopathic and non-idiopathic patients. Thus, only the maps containing high-signal voxels (one map per neuroimage) were used in the subsequent analyses.

### 2.5. Intensity normalization based on the gaussian distribution

**Figure 2:**
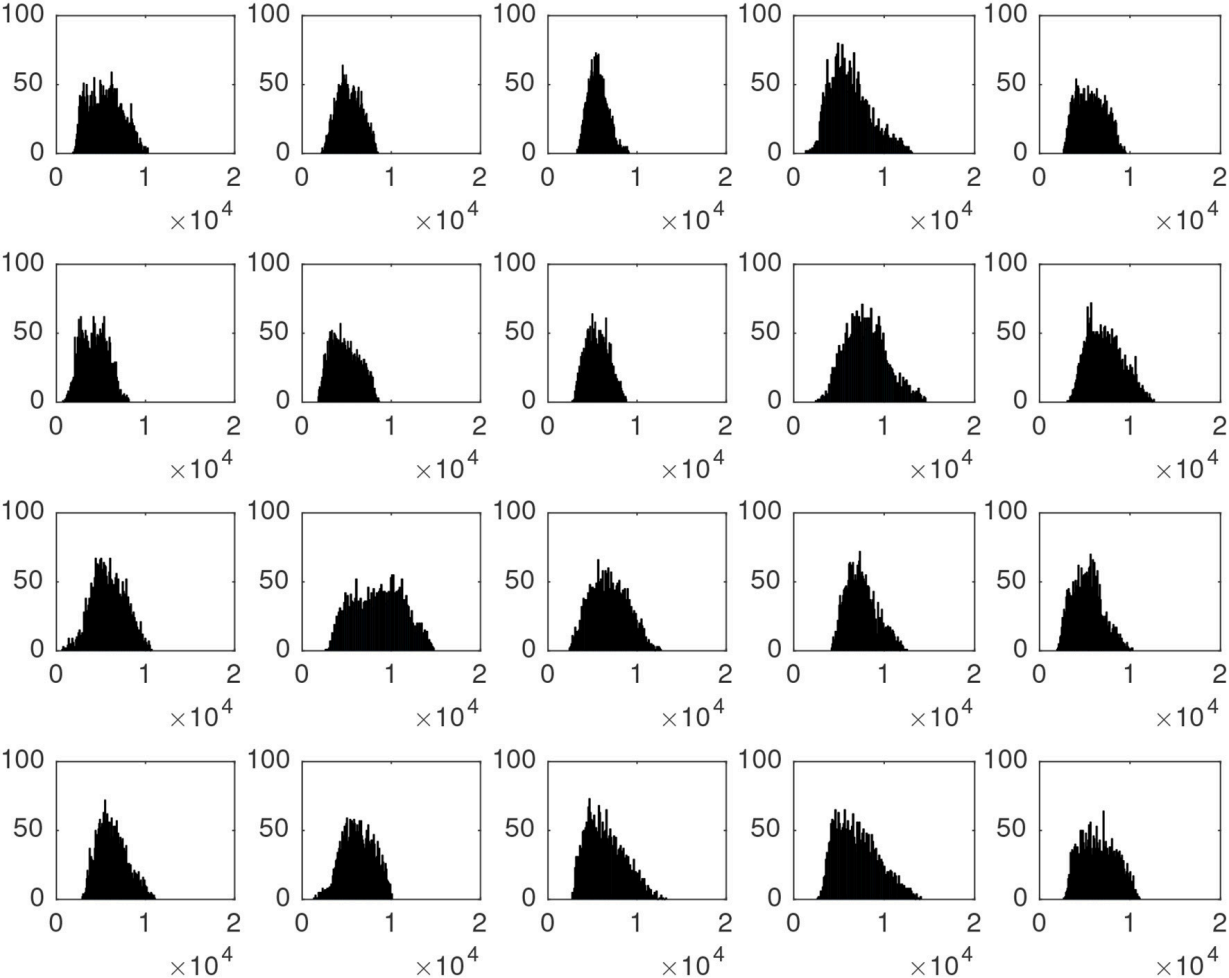
Histograms associated to the striatal voxels of the first 20 neuroimages in our dataset. All of them correspond to idiopathic parkinsonian patients.

The intensity of high-signal voxels largely differs from one patient to another, even among patients suffering the same parkinsonian disorder. This can be noted on Figure 2, which shows the histogram of the map containing these voxels for the first 20 patients in our dataset (all of them were diagnosed with idiopathic parkinsonim).

In order to reduce these differences without losing the discriminant information contained in the data, an additional normalization step was performed. This procedure modeled the histogram of a given map of each patient by a Gaussian distribution. Then, these data were modified so that the Gaussians corresponding to all the patients have approximately same mean and standard deviation. First, parameters *G*_*μ*_ and *G*_*σ*_ were computed:

(10) $$\begin{array}{rcl} G_{\mu } & = & \frac{1}{n}\overset{n}{\underset{i\;=\;1}{\sum }}\mu_{{\text{p}}_{i}} \end{array}$$

(11) $$\begin{array}{rcl} G_{\sigma } & = & \frac{1}{n}\overset{n}{\underset{i\;=\;1}{\sum }}\sigma_{{\text{p}}_{i}} \end{array}$$

where *μ*_**p**_*i*__ and *σ*_**p**_*i*__ respectively stand for the mean and standard deviation of the Gaussian associated to data from patient **p**_*i*_, and *n* is number of patients/neuroimages in our dataset. The data from each patient were then modified as follows:

(12) $$\begin{array}{r} {\text{p}}_{i}^{\left(NORM\right)}=G_{\sigma }\frac{{\text{p}}_{i}-\mu_{{\text{p}}_{i}}}{\sigma_{{\text{p}}_{i}}}+G_{\mu } \end{array}$$

**Figure 3:**
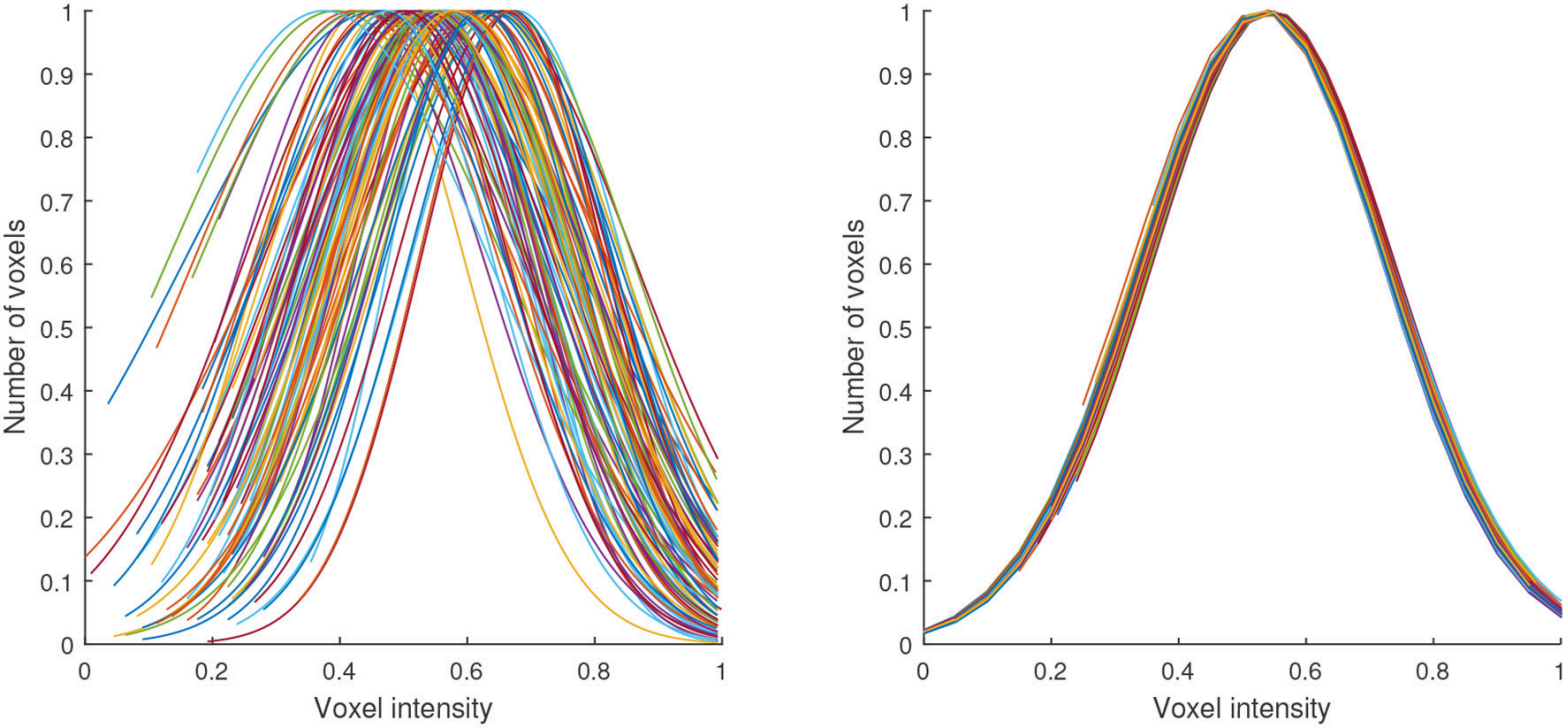
Gaussian distributions modeling the histograms of the maps with high-signal voxels from all the neuroimages in our database before (left) and after (right) the proposed intensity normalization. Note that after normalization the histogram corresponding to all the maps can be modeled by a Gaussian with the same shape.

Figure 3 illustrates the transformation carried out by this procedure. It shows the shape of the histograms of our data before and after the normalization. Note that this procedure was independently applied to the data of each patient and in our case, it was only used to normalize the maps with high-signal voxels, however it can be also applied other maps obtained from the segmentation.

## 3. Results and discussion

In order to evaluate the advantages of preprocessing ^18^F-DMFP-PET data with our methodology, a statistical classification analysis was carried out. To this purpose, a SVM classifier (Vapnik, 2000) was used after the preprocessing steps to separate the idiopathic and non-idiopathic patients in our database (i.e., PD vs. MSA and PSP). As it is common in PD diagnosis (Winogrodzka et al., 2001; Constantinescu et al., 2011; Niccolini et al., 2014; Prashanth et al., 2014) we used only the voxels at the striatum, as selected by the maps with high-signal voxels (the other maps resulting from the segmentation were discarded). The normalized intensity values of the selected voxels were directly used as feature.

The classification performance was estimated by means of a *k*-fold cross-validation scheme (*k* = 5). In order to avoid biased results, all the parameters required by the method were fit inside the cross-validation loop, using only the training data. A nested loop was also used to adjust the parameter *C* of the SVM classifier (Varma and Simon, 2006). Table 2 shows the achieved accuracy, sensitivity and specificity (idiopathic patients were considered as positive) and compares these results with the ones obtained by other approaches: (i) selecting voxels at the striatum by means of an atlas and, (ii) using all the voxels of the brain. In these cases, the intensity of the voxels was normalized using the normalization to the maximum (Saxena et al., 1998).

**Table 2:** Accuracy, sensitivity and specificity obtained by a SVM classifier when separating idiopathic and non-idiopathic Parkinsonism.

**Features set**	**Accuracy (%)**	**Sensitivity (%)**	**Specificity (%)**
Striatum (proposed method)	75.86	74.36	77.08
Striatum (atlas)	72.41	66.67	77.08
All the voxels	65.52	56.41	72.92

The results shown in Table 2 suggest that our preprocessing method allows improving the automatic separation of parkinsonian patients. The relatively low rates achieved by the SVM classifier are due to the dataset used in this work. Most of the neuroimages correspond to patients in a very initial stage. In fact they were acquired 2 years before obtaining the final diagnosis used to label the data. In addition, the whole brain approach also suffers from the small sample size problem (Duin, 2000). In this classification the number of features is larger and many of these features correspond to regions of low signal in ^18^F-DMFP-PET data, which are not useful to separate the groups. In terms of sensitivity and specificity, the obtained results show that the proposed method largely improve (about 8%) the ability of the classifier to correctly detect the positive subjects (idiopathic Parkinsonism) however the improvement in the true negative rate is limited, specially when compared with the atlas-based approach. This fact can be explained by the heterogeneity of the negative group (composed by subjects diagnosed with MSA and PSP), which makes more difficult to characterize the data.

As mentioned above, the analysis of neuroimaging data for diagnostic purposes in PD-related studies is commonly focused on the striatum. In fact, post-mortem studies reveled that most of the neuropathological hallmarks of PD are gathered in this area (Rinne et al., 1991; Hartmann, 2004; Nagatsu and Sawada, 2007). Nonetheless the region to be analyzed highly depends on the neuroimage modality or, more specifically, on the binding properties of the radiotracer used. For example, studies using ^123^I-FP-CIT (Winogrodzka et al., 2001; Spiegel et al., 2007) frequently constraint their analyses to the striatum, since this radiotracer binds to dopamine transporters, whereas studies based on ^18^F-FDG usually analyze the whole brain (Hellwig et al., 2012; Garraux et al., 2013) since this drug measures the brain metabolism. ^18^F-DMFP is commonly used to study the striatal dopamine (Schreckenberger et al., 2004) and indeed, the vast majority of high-intensity voxels in ^18^F-DMFP-PET images are gathered in the striatum. However, these data show a not insignificant part of the total intensity in regions other than the striatum (Segovia et al., 2017). The segmentation methodology proposed in this work allows scientists to independently analyze high-signal and medium-signal voxels (respectively located in the striatum and in the remaining regions in ^18^F-DMFP-PET data) while low-signal voxels (with low signal-noise ratio) are discarded.

Compared with an atlas-based approach, our segmentation method not only provides a higher accuracy in the subsequent classification procedure but also allows the separation of regions of interest in the image space. Thus, it is not necessary to transform the data to the atlas space, avoiding the distortions introduced by these procedures (Ashburner and Friston, 2007).

**Figure 4:**
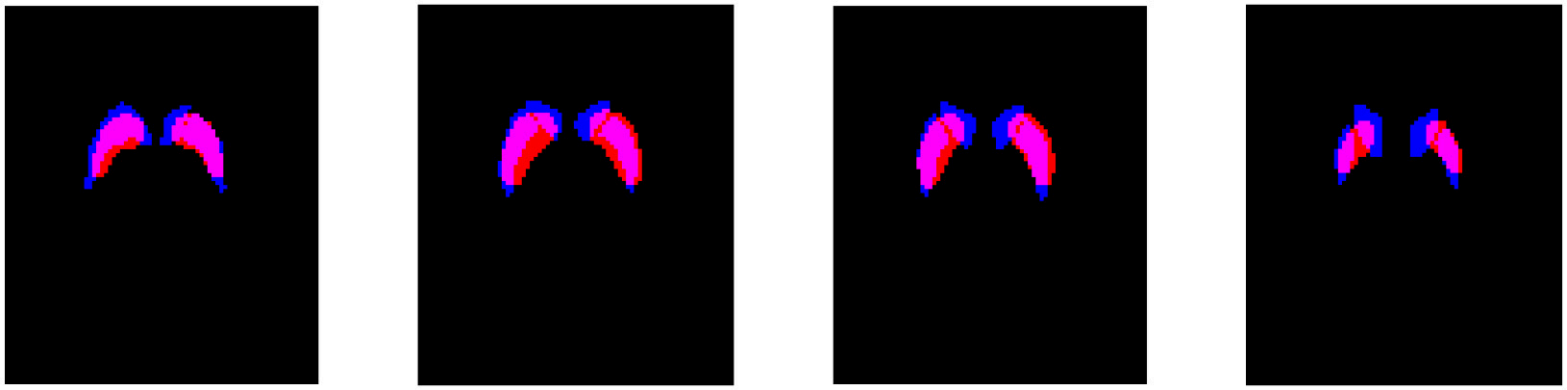
Overlap of the striatum mask obtained by the HMRF-based segmentation (red) and the atlas-based approach (blue). Four axial slices located respectively at −6, 0, 6, and 12 mm from the anterior commissure are shown.

A comparison between the striatum region obtained by the HMRF-based segmentation method and the atlas-based approach is shown in Figure 4. A quantitative analysis of this comparison reveals that: (i) the striatum region is about 30% larger when obtained by means of the atlas-based approach; (ii) most of the voxels selected by the proposed method (about 72%) were also selected by the other approach. Thus, the improvements in the classification procedure are probably because the HMRF-based segmentation provide a more accurate delimitation of the discriminant voxels. Most of these voxels are located in the striatum but not all the voxels in the striatum should be considered to separate idiopathic and non-idiopathic Parkinsonism. According to the results shown in Table 2, discarding these moderately discriminant voxels of the striatum provides larger sensitivity rates but have a reduced impact in the specificity.

**Figure 5:**
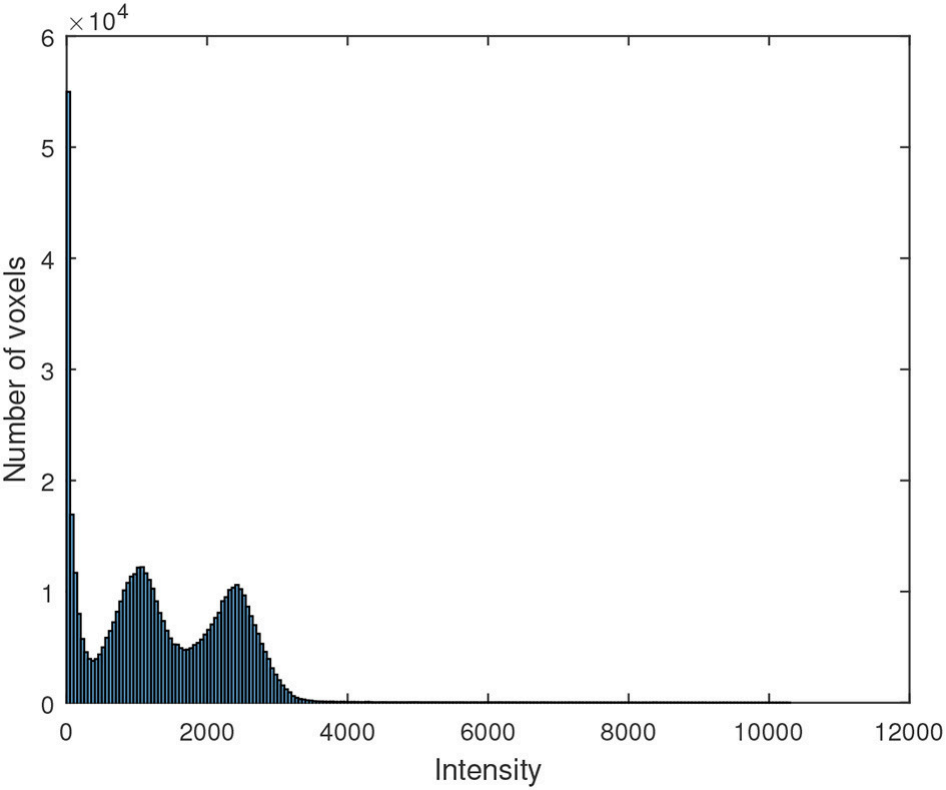
Histogram of a ^18^F-DMFP-PET neuroimage corresponding to a patient diagnosed with idiopathic Parkinsonism.

The motivation to use Gaussian distributions to model the histogram of the maps resulting from the segmentation is explained by the Figure 5, which shows the histogram of a ^18^F-DMFP-PET neuroimage. The two Gaussians corresponding to maps with the low-signal and medium-signal-voxels can be clearly identified. The Gaussian for high-signal voxels has much less height than the remaining ones and can not be appreciated in Figure 5 but it can be identified in the histograms of Figure 2. Finally, the voxels with intensity very close to zero could be modeled by a fourth Gaussian. Indeed, the segmentation of a ^18^F-DMFP-PET neuroimage using this algorithm is similar to model the histogram of that neuroimage by a sum (or mixture) of 4 Gaussians (Segovia et al., 2010; Górriz et al., 2011). Nevertheless, the HMRF approach takes into account both, the voxel intensity and the voxel neighborhood to associate each voxel to a specific map/Gaussian.

In this work, the segmentation method was applied only to an average neuroimage and the result was used to parcel each individual neuroimage. This approach requires lower computational burden than the straightforward alternative consisting on applying the segmentation algorithm to each neuroimage. Additionally the resulting maps are of equal size for all the neuroimages, what allows us to directly use the voxels as feature in the subsequent classification step.

## 4. Conclusion

In this manuscript we described a novel methodology to preprocess ^18^F-DMFP-PET data in order to improve the diagnosis of Parkinsonism. The preprocessing method was carried out in two steps. First, using a HMRF-based approach, each neuroimage was divided into 4 maps according to the intensity and the neighborhood of the voxels. Then, the intensity of the voxels was normalized using the properties of the Gaussian distribution. To this end, the histogram of each map was modeled by a Gaussian distribution with the same parameters for all the neuroimages.

This methodology was evaluated using a dataset with neuroimaging data from 87 patients diagnosed with idiopathic or non-idiopathic Parkinsonism. Using the proposed methodology, we selected and normalized the high-signal voxels of each neuroimage. These data were used to train a SVM classifier in order to separate idiopathic and non-idiopathic subjects, obtaining an accuracy rate about 75%. These results outperform those reported by previous approaches, what suggests that our preprocessing method improves the computer tools currently used to assist the diagnosis of Parkinsonism.

## Author contributions

Drafting the article and conception or design of the work: FS. Critical revision of the article, data analysis and interpretation: FS, JG, JR, FM, and DS.

### Conflict of interest statement

The authors declare that the research was conducted in the absence of any commercial or financial relationships that could be construed as a potential conflict of interest.
